# Genome-wide analysis of *CsWOX* transcription factor gene family in cucumber (*Cucumis sativus* L.)

**DOI:** 10.1038/s41598-020-63197-z

**Published:** 2020-04-10

**Authors:** Ran Gu, Xiaofei Song, Xiaofeng Liu, Liying Yan, Zhaoyang Zhou, Xiaolan Zhang

**Affiliations:** 1 State Key Laboratories of Agrobiotechnology, Beijing Key Laboratory of Growth and Developmental Regulation for Protected Vegetable Crops, MOE Joint Laboratory for International Cooperation in Crop Molecular Breeding, China Agricultural University, Beijing, 100193 China; 2 Analysis and Testing Centre, Hebei Normal University of Science & Technology, Qinhuangdao, 066004 China; 3 College of Horticulture Science and Technology, Hebei Normal University of Science& Technology, Qinhuangdao, 066004 China

**Keywords:** Developmental biology, Plant sciences

## Abstract

WUSCHEL-related homeobox (WOX) transcription factors are plant-specific members that characterized by the presence of a homeodomain. It has been shown that WOX members regulate several aspects of plant development, but the biological functions of this *CsWOX* gene family remain largely unknown in cucumber (*Cucumis sativus* L.). In this study, we identified and characterized 11 putative *CsWOX* genes in cucumber, which are also divided into three major clades (e.g., the Ancient clade, the Intermediate clade and the WUS clade). Expression pattern analysis revealed tissue-specific expression patterns of *CsWOX* genes, including that *CsWOX9* is mainly expressed in developing fruit and also has lower expression in tip and axillary bud, which was further confirmed by *in situ* hybridization assay. Moreover, overexpression of *CsWOX9* in Arabidopsis led to increased branches and rosette leaves, and shorter siliques. Together, these results indicated that CsWOX members may regulate cucumber growth and development.

## Introduction

WUSCHEL-related homeobox (WOX) gene family are plant-specific transcription factors, which belong to homeobox superfamily. All WOX members contain a homeodomain with 60–66 amino acid residues. According to evolutionary relationships, they can be divided into WUS, Intermediate and Ancient clades^1–3^. These WOX members have been identified and studied in many species including Arabidopsis, rice, maize and soybean.

So far, it has been found that WOX transcription factors play important roles in regulating many aspects of growth and development including meristematic stem cell maintenance, embryonic development and polarization, lateral organ development, and organ regeneration. The founding member *AtWUS* is expressed in stem apical meristem (SAM) and is necessary for SAM formation and maintenance^2,4–6^. In addition to AtWUS, other WOX members are also involved in stem cell maintenance. For example, *AtWOX5* is enriched in the quiescent center and plays an important role in maintaining the stability of stem cells of root apical meristem, similar to AtWUS in SAM^7^. *WOX4* is mainly expressed in the procambium and cambium, and promotes differentiation and/or maintenance of the vascular procambium in Arabidopsis and tomato^8,9^. WOX9 promotes the growth of SAM and is required for the maintenance of *WUS* expression at the shoot apex, and mediates cytokinin signaling during shoot meristem establishment^10^.

The involvement of WOX members in plant development is not limited to stem cell maintenance. For example, AtWOX2 contributes to early pre-embryo and cotyledon boundary formation during embryo development in the egg cell and zygote^11^. Consistently, PaWOX2 also likely regulates embryo cell division and/or differentiation in *Picea abies*^12^. *AtWOX7* is expressed at all stages of lateral root development, and inhibits lateral root development in a sugar-dependent manner^13^. Similarly, *OsWOX6* and *OsWOX11* are expressed asymmetrically in response to auxin control rice tiller angle^14^. Moreover, AtWOX11 and AtWOX12 play important roles in de novo root organogenesis. In responds to a wounding-induced auxin maximum, AtWOX11 and AtWOX12 promote the first-step cell fate transition from a leaf procambium to a root founder cell, initiating adventitious root organogenesis^15^. It has been reported that *AtWOX13* is mainly expressed in meristematic tissues including the replum and promotes replum development and fruit opening^16,17^.

Cucumber (*Cucumis sativus* L.) is one of the important economic vegetable crops. Many growth and development processes affect cucumber quality and yield. Although WOX members have been shown to regulate many aspects of development in several plant species, only CsWOX1 was reported to regulate early reproductive organ development in cucumber^18^. It is unknown whether CsWOX members regulate different biological processes in cucumber. Here, we identified and characterized 11 putative *CsWOX* genes. To investigate the potential functions of *CsWOX* genes in cucumber, gene expression analysis showed that these *CsWOX* genes have different tissue-specific expression patterns. When introduced *CsWOX9* driven by the *35S* promoter into the Arabidopsis, the transgenic lines exhibited shorter siliques and increased branches, consistent with its expression pattern in cucumber. Together, these results indicated that CsWOX members may regulate development in cucumber.

## Materials and methods

### Plant materials

Cucumber (*Cucumis stativu*s L.) inbred R461 were grown in a standard glass greenhouse of China Agricultural University (Beijing, China). Irrigation and pest control were carried out according to standard procedures. The Arabidopsis Columbia (*Col*) was used as wild-type (WT) and grown at 22 °C in a greenhouse with a 16-h-light/8-h-dark photoperiod.

### Gene identification

To identify *CsWOX* genes in the cucumber genome (http://cucurbitgenomics.org), the full-length sequences of 15 AtWOX members obtained from Arabidopsis genome database (https://www.arabidopsis.org) were used as queries for BLASTp searches. Subsequently, the sequences of the candidate members were analyzed using SMART (http://smart.embl-heidelberg.de) and Pfam (http://pfam.xfam.org) to exclude the members that did not contain the complete homeodomain.

### Bioinformatics analysis of CsWOX family members

The amino acid sequences of CsWOX members in cucumber were obtained from the Cucumber Genome Database (http://cucurbitgenomics.org/), and then the molecular weight and isoelectric points of these members were analyzed using ExPASy Proteomics Server (https://web.expasy.org/protparam/). The gene structure analysis was performed using the online program Gene Structure Display Server (GSDS: http://gsds.cbi.pku.edu.cn)^19^. The *cis*-acting elements in the promoters of *CsWOX* genes were analyzed using the PlantCARE (http://bioinformatics.psb.ugent.be/webtools/plantcare/html/)^20^.

The full-length sequences of CsWOX family members were aligned using ClustalW in MEGA 5.0 and this alignment was used to generate the phylogenetic tree with Neighbor-Joining (NJ) method. Poisson correction, pairwise deletion and a bootstrap were conducted with 1000 replicates^21^.

### Gene expression analysis

Total RNA was extracted using a Quick RNA Isolation Kit (Waryoung, Beijing, China) and cDNAs were synthesized using TianScript II RT Kit (Tiangen Biotech, China). The qRT-PCR analysis was performed using ABI PRISM 7500 Real-Time PCR System (Applied Biosystems). The cucumber *UBIQUITIN* (Csa000874) and Arabidopsis *ACTIN2* (AT3G18780) genes were used as internal controls. Three biological replicates were performed. Sequences of all the primers used in this study were shown in Table S2.

For gene expression pattern analysis, the cucumber root, stem, leaf, tip and tendril of 16-day-old seedlings, axillary bud from the top of the lateral branch, male and female buds at the 10^th^ stage of flower development, male and female flowers at anthesis, 1.5 cm fruit, 2 cm fruit and fruit on the day of blooming were harvested for RNA isolation.

For analysis of ABA and auxin-induced gene expression, the two-week-old cucumber seedlings were treated with 100 μM abscisic acid (ABA) or 100 μM indole-3-acetic acid (IAA). The seedling tips were collected at 0, 0.5, 1, 3, 6, 9, 12 and 24 h after treatment.

### *In situ* hybridization

The shoot tips of 16-, 18-, and 20-day-old seedlings, young floral buds and young fruits were fixed in 3.7% FAA (3.7% formaldehyde, 5% glacial acetic acid, and 50% ethanol)^22^, and then embedded, sectioned and hybridized with digoxigenin (DIG)-labeled sense and antisense gene-specific probes as previous described^23^. The primer information for probe was listed in Table S2.

### Arabidopsis transformation

To generate the *CsWOX9* overexpression transgenic plants, the *CsWOX9* coding sequence was cloned into the pBI121 vector. The resultant construct was introduced into *Agrobacterium* strain C58 by electroporation and transformed into Arabidopsis plants using the floral-dip method^24^. Primary transformants were screened on Murashige and Skoog (MS) medium with 40 mg·L^−1^ kanamycin. The primers used for vector construction were listed in Table S2.

## Results

### Identification and phylogenetic analysis of *CsWOX* genes in cucumber

To identify CsWOX members in cucumber, we performed BLASTp searches using the full-length sequences of 15 Arabidopsis AtWOX members and analyzed homeodomain using SMART software^11^. Consistent with previous study^18^, 11 putative *CsWOX* genes were identified in cucumber (Table 1). These genes encoded proteins ranging from 130 and 387AA (amino acids) while isoelectric point (pI) values ranging from 5.54 to 10.08, and the molecular weights from 14.66 to 43.64 kDa (Table 1).

**Table 1:** Characteristics of CsWOX transcription factors in cucumber.

Name	Gene ID	Genomic Location	Clade	AA	MW (kDa)	pI
*CsWUS*	Csa6G505860	25736729..25738425	WUS Clade	304	33.41	6.25
*CsWOX1a*	Csa1G042780	4494646..4497727	WUS Clade	387	43.64	5.76
*CsWOX1b*	Csa1G025040	2675438..2678242	WUS Clade	334	39.12	8.49
*CsWOX2*	Csa1G505930	17913741..17915174	WUS Clade	239	26.88	6.66
*CsWOX3*	Csa6G301060	14353366..14354731	WUS Clade	193	22.37	6.45
*CsWOX4*	Csa2G356610	16722291..16723235	WUS Clade	227	25.88	8.94
*CsWOX7*	Csa6G010010	1117959..1118864	WUS Clade	130	14.66	10.08
*CsWOX9*	Csa6G518270	27456068..27458240	Intermediate Clade	376	41.11	8.33
*CsWOX11*	Csa3G812740	31266731..31269991	Intermediate Clade	257	27.33	5.97
*CsWOX13a*	Csa3G002330	249427..252960	Ancient Clade	282	32.11	6.00
*CsWOX13b*	Csa4G663700	23044894..23046632	Ancient Clade	269	30.64	5.54

**Figure 1:**
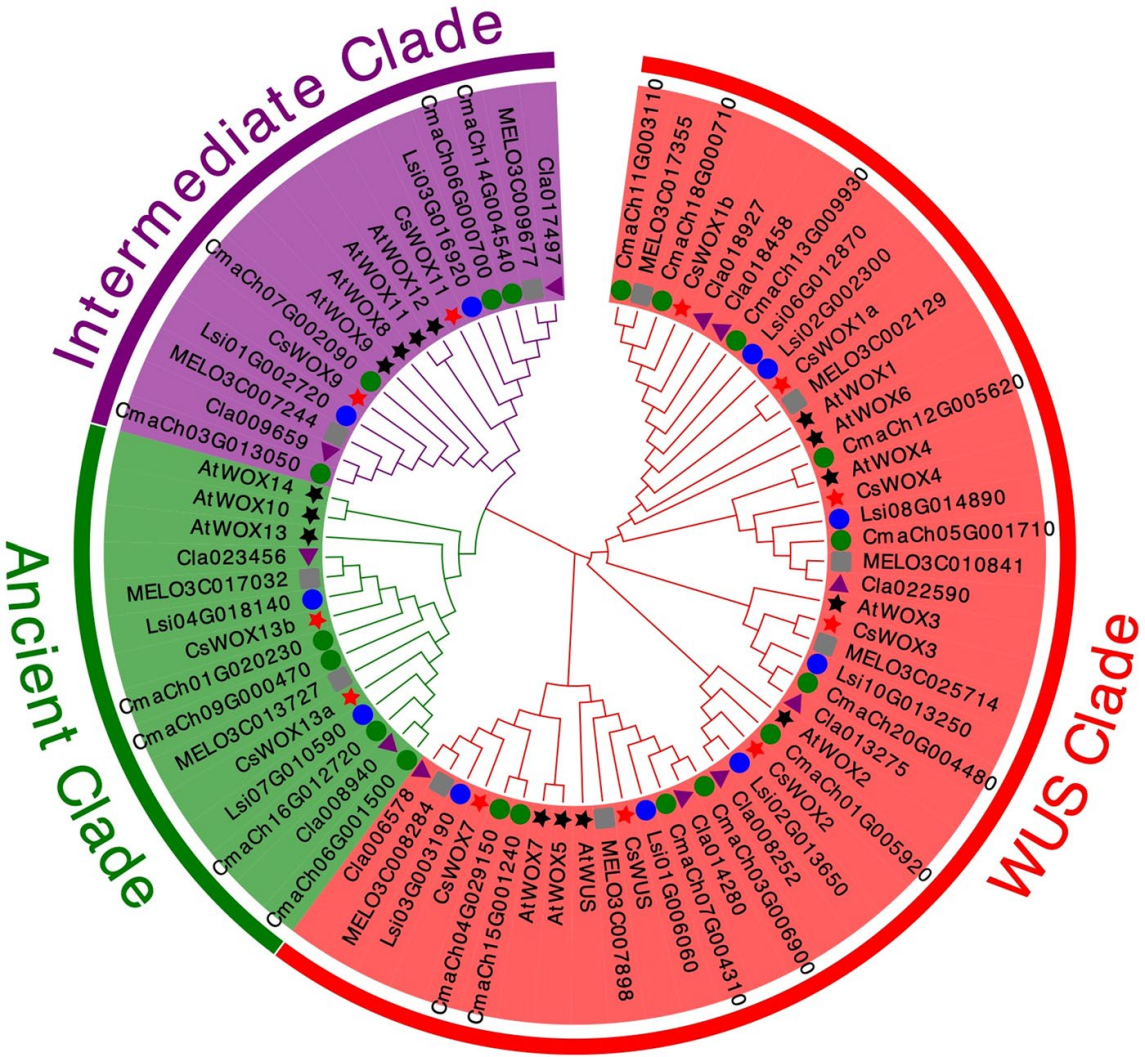
Phylogenetic clustering of WOX members. The full-length sequences of WOX members were used to generate the phylogenetic tree using MEGA. Arabidopsis (15 genes), cucumber (11 genes), melon (10 genes), watermelon (11 genes), pumpkin (19 genes) and calabash (11 genes) are indicated with black stars, red stars, grey rectangles, purple triangles, green circles and blue circles, respectively. Each of the three WOX clades is indicated in a specific color.

**Figure 2:**
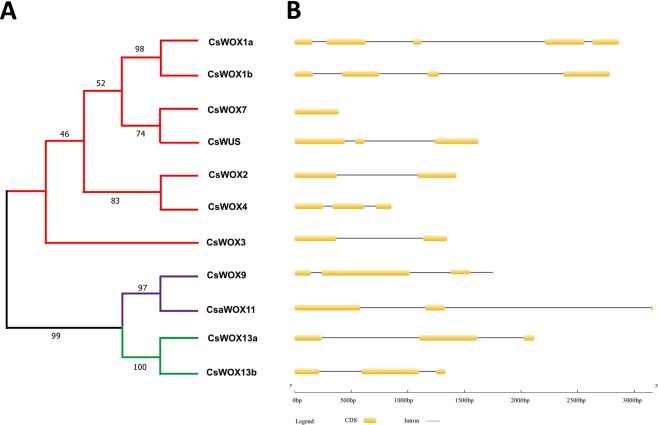
Phylogenetic clustering and gene structures of CsWOX members in cucumber. (**A**) The phylogenetic tree was generated based on the full-length sequences of cucumber CsWOX proteins. WUS Clade, Intermediate Clade and Ancient Clade are indicated in red, purple and green colors, respectively. (**B**) Exon-intron distribution of cucumber CsWOX genes. Yellow boxes indicate exons and black lines indicate introns.

To explore the evolutionary relationship of cucurbit and Arabidopsis WOX members, the full-length sequences of these members were used to generate the phylogenetic tree using MEGA5.0. These cucurbit members were renamed according to the original Arabidopsis names clustered in the same clade. The WOX members in every species can be divided into three clades, which were the Ancient clade, Intermediate clade and WUS clade. The WUS clade was the largest one in the tree and also remained the largest group in Arabidopsis and each cucurbit. While the Ancient clade only contains 15 WOX members and is the smallest clade in each tested species (Figs. 1 and 2A), indicating that WOX members between Arabidopsis and cucurbit species may have a close evolutionary relationship.

### CsWOX gene structures and conserved domains

To better understand the *CsWOX* gene structure in cucumber, the exon/intron organization was analyzed. The results showed that all *CsWOX* genes contain one to five exons (Fig. 2B). Interestingly, both *CsWOX13a*/*13b* in the Ancient clade has three exons. The exon number of *CsWOX* genes is variable in WUS and Intermediate clades. For example, *CsWOX1a* contains five exons, while *CsWOX7* only has a single exon (Fig. 2B).

**Figure 3:**
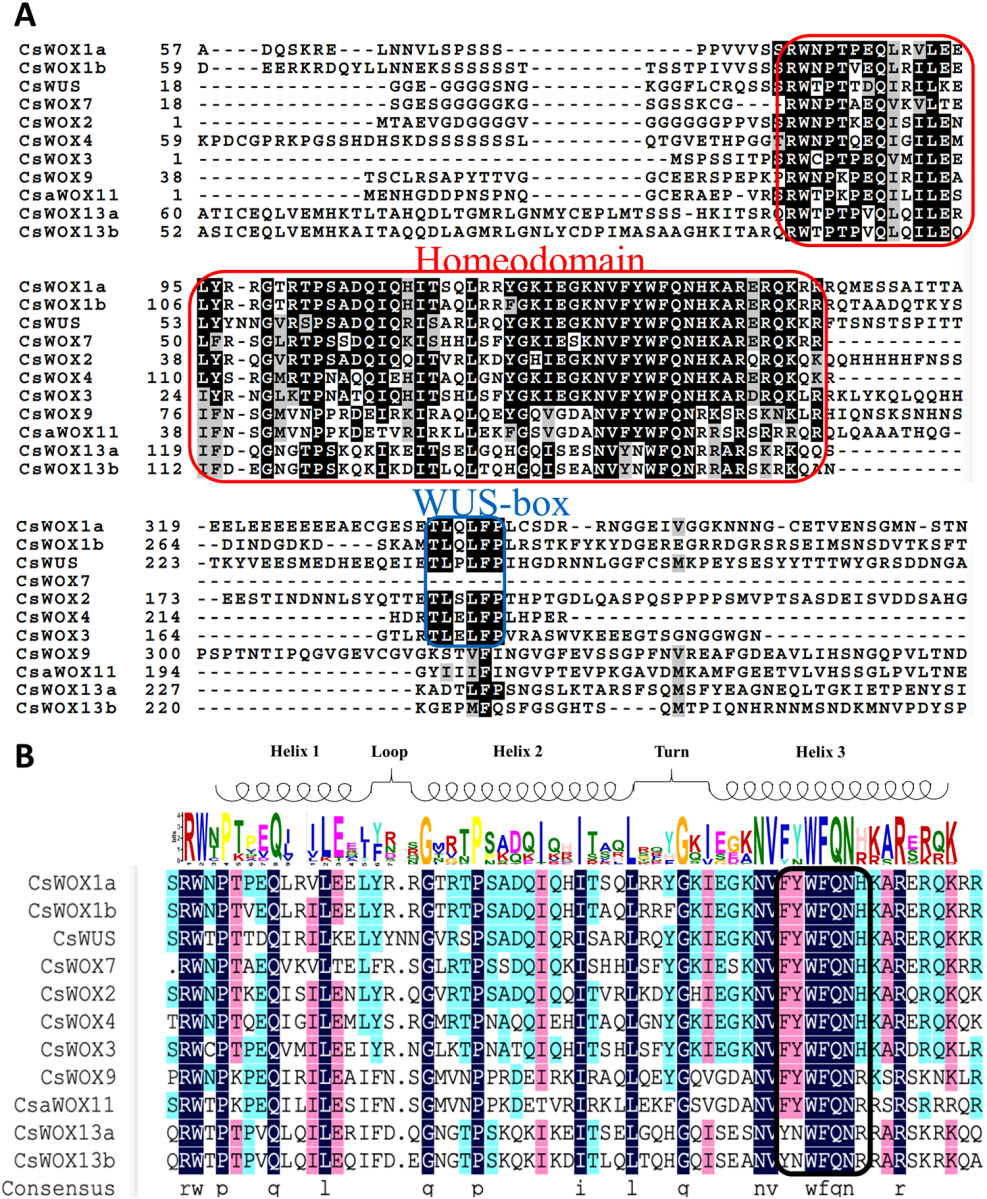
Protein sequence and conserved homeodomain analysis of CsWOX family members. (**A**) The sequence alignment of CsWOX members in cucumber. The red box indicates homeodomain and the blue box represents WUS-box domain. (**B**) Sequence alignment of homeodomain of CsWOX proteins in cucumber. The alignment was colored according to percent identity.

The WOX members are plant-specific proteins containing a conserved homeodomain^25,26^. To study whether the domain is conserved in CsWOX members, the sequences of these proteins were aligned to generate sequence logos. The alignment results showed that all the CsWOX members contain a conserved homeodomain that carrying a helix-loop-helix-turn-helix structure (Fig. 3). Consistent with previous studies^27,28^, the representative motifs **YN**WFQN**R**, **FY**WFQN**R** and **FY**WFQN**H** also exist in homeodomains from the Ancient, Intermediate and WUS clades, respectively (Fig. 3B). Moreover, the CsWOX members in WUS clade also contain a WUS-box, except CsWOX7 (Fig. 3A). While CsWOX members in Ancient and Intermediate clades have no WUS-box, indicating that these CsWOX members in different clades may be involved in different biological processes in cucumber.

### Expression pattern of *CsWOXs* in cucumber

**Figure 4:**
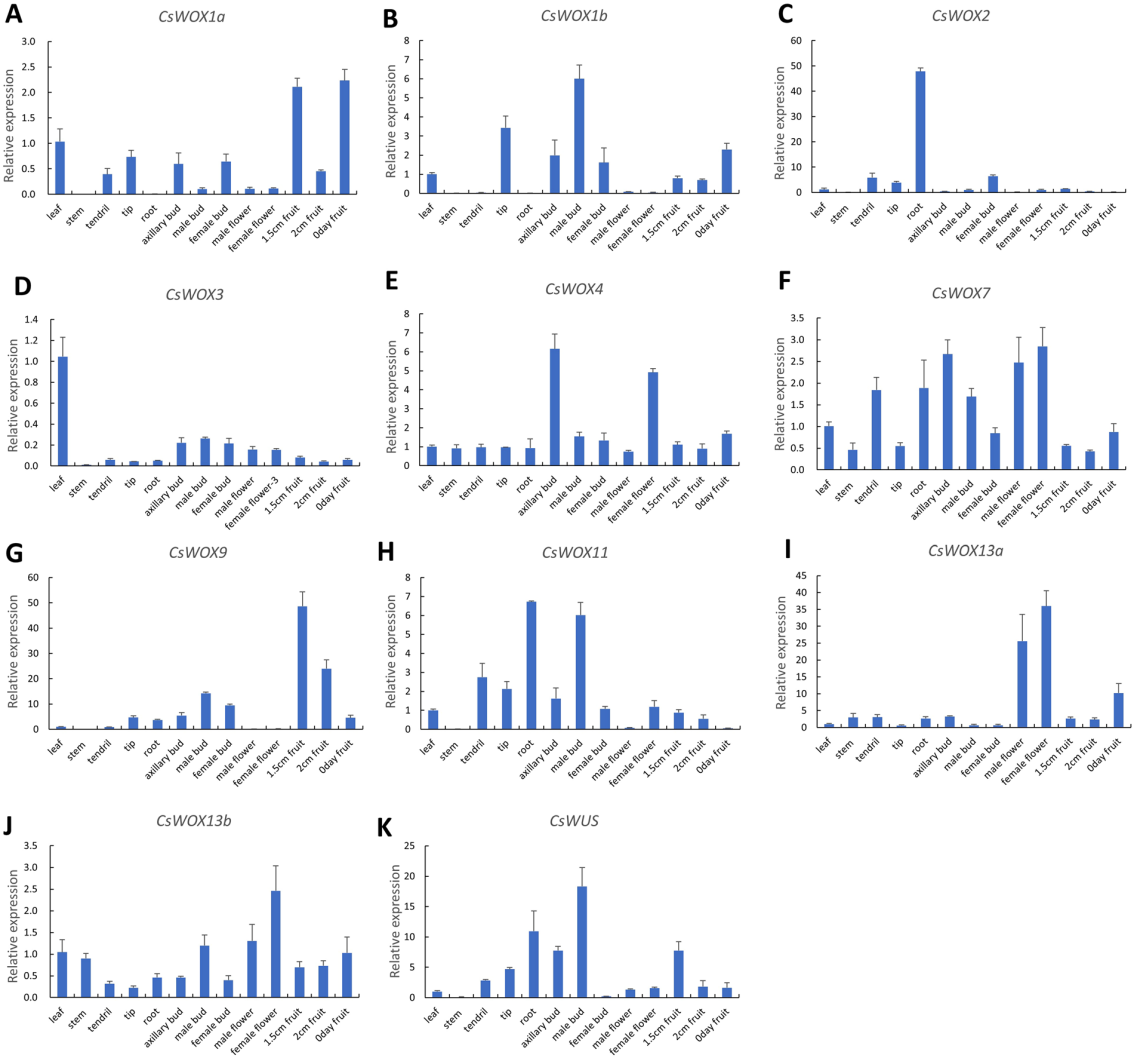
Expression patterns of *CsWOX* genes in cucumber. (**A**‒**K**) Tissue-specific of *CsWOX* expression in cucumber was examined using qRT-PCR. The cucumber *UBIQUITIN* gene was used as an internal standard. Values are means ± sd of three biological replicates.

To investigate the details of tissue-specific expression of the *CsWOX* genes in cucumber, the expression patterns of all 11 *CsWOX* genes were examined by quantitative RT-PCR (qRT-PCR) in thirteen tissues/organs, i.e., leaf, stem, tendril, tip, axillary bud, root, male bud, female bud, male flower, female flower, 1.5 cm fruit, 2 cm fruit and fruit on the day of blooming (0 day fruit). The results showed that *CsWOX2* was mainly expressed in root; *CsWOX3* was specifically expressed in leaf; *CsWOX4* was specifically enriched in axillary bud and female flower; and *CsWOX13a* was highly accumulated in male and female flowers, while *CsWOX1a, CsWOX1b, CsWOX7, CsWOX11, CsWOX13b* and *CsWUS* showed no obvious organ-specific expression patterns (Fig. 4). Interestingly, *CsWOX9* was mainly expressed in different developmental stages of fruit and also has lower expression in tip and axillary bud (Fig. 4G). These results suggested that CsWOX members may function in many aspects of cucumber growth and development.

### *CsWOX* gene expression in response to different hormonal treatments

**Figure 5:**
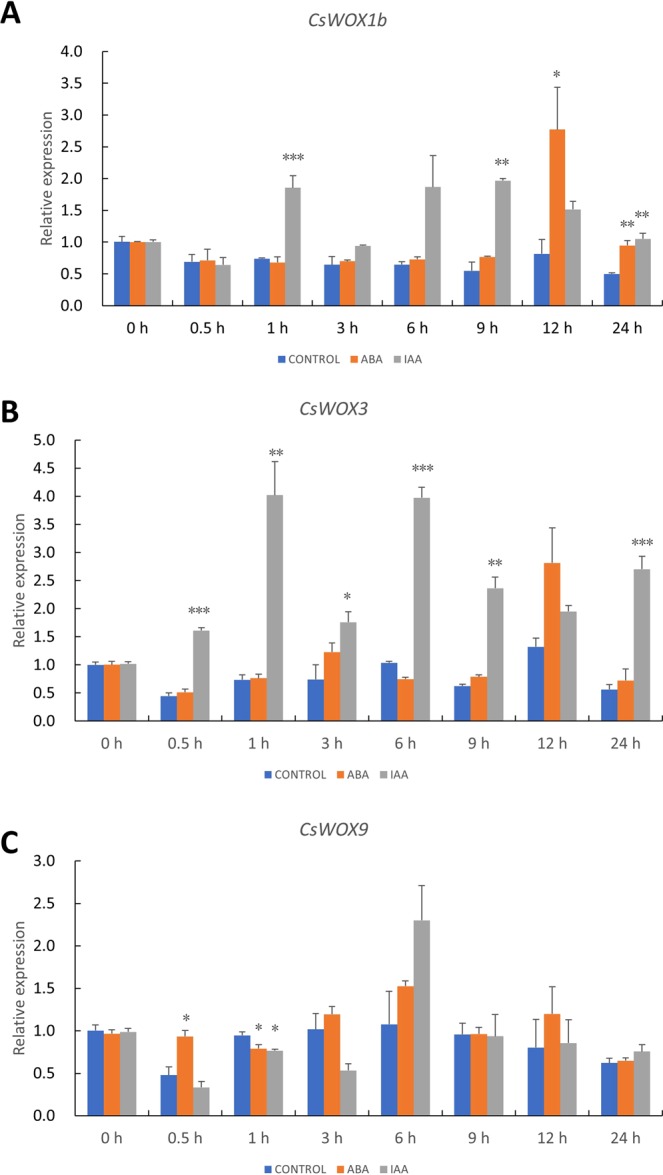
Hormonal regulation of *CsWOX* expression. Two-week-old cucumber seedlings were treated with 100 μM ABA or 100 μM IAA. The expressions of *CsWOX1b* (**A**), *CsWOX3* (**B**) and *CsWOX9* (**C**) were examined at 0 h, 0.5 h, 1 h, 3 h, 6 h, 9 h, 12 h and 24 h after treatment. Values are means ± sd of three biological replicates, and significant differences between the treated seedlings and control are indicated by asterisks (*P < 0.05 and **P < 0.01, ***P < 0.001, one-way ANOVA, Tukey post-test, three independent experiment).

Plant hormones have been shown to regulate plant development. To investigate whether *CsWOX* genes are regulated by plant hormones, the promoter sequences (2000 bp upstream of the start codon) of *CsWOXs* were analyzed by PlantCARE. Many *cis*-elements were detected in the *CsWOX* gene promoter regions including some respond to auxin, abscisic acid (ABA), gibberellin (GA), salicylic acid (SA) and Methyl jasmonate (MeJA) (Table S1). Previous studies showed that auxin and ABA regulated WOX expression in rice and *Cunninghamia lanceolate*^29,30^. To further confirm that the expression of *CsWOX* genes is also regulated by plant hormones, these members (*CsWOX1b, CsWOX3* and *CsWOX9*) carrying auxin or/and ABA response elements in their promoters were selected for study. As expected, our results showed that the expression of *CsWOX1b* and *CsWOX3* could be greatly increased by indole-3-acetic acid (IAA) treatment, and the transcript levels of *CsWOX1b* and *CsWOX9* could respond rapidly to ABA treatment (Fig. 5). Together, these results suggested that some CsWOX members may be involved in different hormone-mediated development.

### Ectopic expression of *CsWOX9* affected plant architecture and fruit length in Arabidopsis

**Figure 6:**
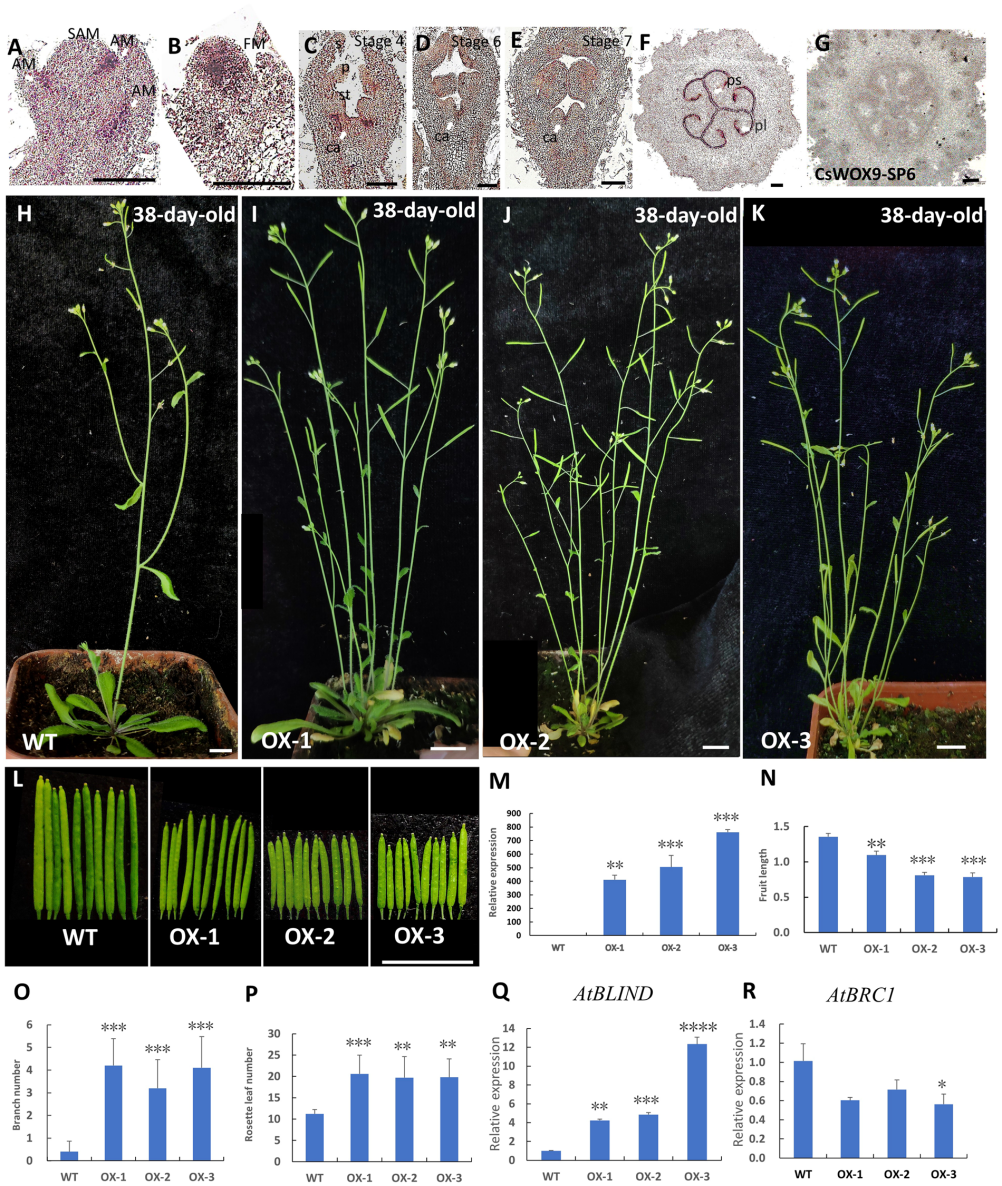
Ectopic expression of *CsWOX9* in the wild type (WT) *Arabidopsis*. (**A-G**) *In situ* hybridization analysis of *CsWOX9*. Negative control of *CsWOX9* hybridized with the sense probe in the fruit (**G**). SAM, Stem Apical Meristem; AM, Axillary Meristem; s, sepal; p, petal; st, stamen, ca, carpel, ps, pseudoseptum; pl, placenta. Scale bar, 100 μm. (**H**‒**K**) Morphology of 38-day-old WT and CsWOX9 transgenic plants. (**L**) Siliques from the primary inflorescence of WT and *CsWOX9* transgenic plants. (**M**) Expression analysis of *CsWOX9* in WT and *CsWOX9* transgenic lines. (**N**‒**P**) Statistical analyses of fruit length (**N**), branch number (**O**) and rosette leaf number (**P**) in WT and *CsWOX9* transgenic plants. (**Q,R**) The expression of *AtBLIND* (**Q**) and *AtBRC1* (**R**) in WT and *CsWOX9* transgenic plants. Values are means ± sd, and significant differences between the transgenic plants and WT are indicated by asterisks (*P < 0.05, **P < 0.01, ***P < 0.001 and ****P < 0.0001, one-way ANOVA, Tukey post-test, three independent experiment).

Given that *CsWOX9* gene was significantly expressed in the developing fruit, it was selected for further study. *in situ* hybridization assay showed that *CsWOX9* mRNA accumulated in the axillary meristem (AM) and the flower meristem (FM) of shoot tips (Fig. 6A,B). *CsWOX9* was also expressed in carpel primordium of flower bud at stage 4, and the joint of adjacent carpals in male flower bud at stage 6 and 7 (Fig. 6C‒E). In young fruit, *CsWOX9* signal was specifically detected in placenta (pl) and pseudoseptum (ps) (Fig. 6F). No signal was detected upon hybridization with the sense *CsWOX9* probe (Fig. 6G).

To investigate the roles of *CsWOX9* in fruit development and other organ development, we further introduced the *CsWOX9* driven by *35S* promoter into wild-type (WT) Arabidopsis. Ten independent transgenic lines were obtained and three representative lines (OX-1, 2, 3) were chosen for further study. All the transgenic plants exhibited shorter siliques (Fig. 6H‒N). The transgenic line OX-3 showed shortest silique lengths compared to other transgenic lines, consistent with their highest expression of *CsWOX9* (Fig. 6L‒N). Interestingly, all the three transgenic plants also displayed increased branches and rosette leaves compared to WT, a result consistent with its expression in axillary bud (Figs. 4G; 6H‒K,O,P). Previous studies showed that *BRANCHED 1* (*BRC1*) inhibits shoot branching, while *BLIND* (*BLIND*) positively regulates axillary meristem formation^31–34^. To further confirm the role of *CsWOX9* in regulating axillary bud outgrowth, we detected the expression levels of *AtBRC1* and *AtBLIND* in *CsWOX9* transgenic plants. As expected, we found that the expression level of *AtBRC1* was strongly reduced and *AtBLIND* was highly increased in three transgenic lines (Fig. 6Q‒R). Taken together, these results support the notion that CsWOX members may regulate many aspects of growth and development in cucumber.

## Discussion

The WOX proteins function in key developmental processes in plants^35–37^. However, only a few of members have been characterized in several plant species. In this study, the full length sequences of AtWOX members were used as queries for BLASTp searches, 11 CsWOX members were identified in cucumber. These CsWOX members can be also classified into three clades (e.g., the Ancient clade, the Intermediate clade and the WUS clade) (Fig. 1). As previous studies^11^, each CsWOX protein contains a homeodomain and all the members in WUS clade have a WUS-box domain except CsWOX7 (Fig. 3A). Interestingly, another previous study also identified the same CsWOX members in cucumber using the homeodomain sequences of AtWOX members for BLASTp searches^18^, indicating that the homeodomain is highly conserved in WOX members.

Expression pattern analysis of these *CsWOX* genes showed that a few of them were mainly expressed in specific organs, indicating that these genes may be involved in different development processes. For example, *CsWOX1a* mutation can lead to a mango fruit in cucumber^18^, which is consistent with that it was mainly enriched in fruit (Fig. 4A). In addition, *CsWOX13a* is accumulated in male and female flowers. Indeed, it has been reported that its homolog AtWOX13 regulated replum development and fruit opening in Arabidopsis^16,17^. But whether CsWOX13a regulates cucumber fruit development remains to be examined.

Our data demonstrated that overexpression of *CsWOX9*, a homolog of *AtWOX9*, resulting in short siliques. This result is consistent with its highest expression in fruit shown by both expression pattern analysis and *in situ* hybridization assay. But whether CsWOX9 is a regulator of fruit development in cucumber remains to be examined. Interestingly, the *CsWOX9* transgenic plants also showed increased rosette leaves and branches, which is expected as its higher expression in axillary bud and axillary meristem shown by qRT-PCR and *in situ* hybridization. It was reported that homologs of WOX8/9 in petunia and tomato plants also contribute to inflorescence architecture by promoting the separation of lateral inflorescence meristems^38,39^. Similarly, *DWARF TILLER1* (*DWT1*), the rice homolog of *AtWOX8/9*, plays important roles in balancing branch growth^40^. These results suggested that the role of WOX8/9 in regulating shoot architecture is conserved in different species. Recent studies showed that auxin plays important roles in regulating fruit length and shoot branching^32,41^. Future studies will need to unravel whether WOX9 controls branch growth by regulating auxin synthesis or/and transport.

## Conclusion

Together, our study identified and characterized 11 CsWOX members in cucumber. Tissue-specific expression of these *CsWOX* genes indicated that they may be involved in different developmental processes. When *CsWOX9* delivered into the Arabidopsis, the transgenic lines exhibited shorter siliques and increased branches. These results uncovered that these CsWOX members play important roles in growth and development.

## Supplementary information

Supplementary Information.
