# Supplementary material for: Genome-wide analysis of CsWOX transcription factor gene family in cucumber (Cucumis sativus L.)
Source: Sci Rep. 2020 Apr 10;10:6216. doi: 10.1038/s41598-020-63197-z (PMC7148364; doi:10.1038/s41598-020-63197-z)
Supplement: Supplementary file 1 — Supplementary Information. [file 41598_2020_63197_MOESM1_ESM.pdf]

Genome-wide analysis of *CsWOX* transcription factor gene family in cucumber (*Cucumis sativus* L.)

Ran Gu, Xiaofei Song, Xiaofeng Liu, Liying Yan, Zhaoyang Zhou*, Xiaolan Zhang*

Table S1. Analysis of hormone regulatory elements of *CsWOX* promoters

Gene	Auxin	ABA	GA	SA	MeJA
CsWUS		2	2		
CsWOX1a	2	1	1		4
CsWOX1b	3	4	1		3
CsWOX2	2	1			3
CsWOX3	1			1	
CsWOX4	1	7	2		1
CsWOX7		3	2		
CsWOX9		1	1	3	1
CsWOX11		1	2		3
CsWOX13a	2	1	1	1	2
CsWOX13b		3		1	4

Table S2. Primers used in this study

Primer name	Primer sequence (5'–3')	Primer name	Primer sequence (5'–3')
**Primers for CsWOX9 amplification and vector construction**			
CsWOX9-clone-F	ATGGCTTCCTCTAACAGACACTGGC	CsWOX9-clone-R	CTATATCAGATAATAGTAAGAACCATGTTGGAGTGAG
wox9bq-pbi121-F	GGACTCTAGAGGATCCATGGCTTCCTCTAACAGACACT	wox9bq-pbi121-R	ACCACCCGGGGATCCCTACAGATCCTCTTCAGAGATGAG
**Primers for qRT-PCR**			
q-CsWUS-F	GGAAATAGCAGTGTCGTCGGG	q-CsWUS-R	ATTATCGTCAGACCGCCCGTA
q-CsWOX1a-F	AAACTGGGTGCCCTCTACAAAC	q-CsWOX1a-R	TTAGTTGGCTACCACCACGGA
q-CsWOX1b-F	CCCAACACCACCATCTACTGCTA	q-CsWOX1b-R	TGGGTCTTCCTTGTAGGGTTTATG
q-CsWOX2-F	AAAGCGGTGGGATTGAGACG	q-CsWOX2-R	CTGCGGGCTGGCTTGTAAAT
q-CsWOX3-F	AGGTGGGTGAAGAGGAGGCA	q-CsWOX3-R	TCCATCTTCCTCCTCCACCG
q-CsWOX4-F	TAAACGCTTACGCCCTCTTGCT	q-CsWOX4-R	CCACAATCGGGTTTGATGAAGG
q-CsWOX7-F	CAGCGGAGAGAGCGGTGGT	q-CsWOX7-R	TCTTGCTCTCAATCTTGCCGTAAA
q-CsWOX9-F	GTCAAGGGTTTTGCTTCTCGGA	q-CsWOX9-R	GTGAGAGGCGTGGAAATAGTGGA
q-CsWOX11-F	GGGGTTGGCGGAGATTGTAG	q-CsWOX11-R	CGAAGGGCAAAACGATGGA
q-CsWOX13a-F	CCTGAGCAGCAAAATCCTTACC	q-CsWOX13a-R	TAGAAGTGATTTTGTGGCTGGAAG
q-CsWOX13b-F	GCCGATGGAGTTGGTGGTC	q-CsWOX13b-R	GTGTAGGCGTCCACCTCTGC
UBI-ep-F	CACCAAGCCCAAGAAGATC	UBI-ep-R	TAAACCTAATCACCACCAGC
q-AT1G55580.1-F	AGAGAGACTAACCCTAGAGCAA	q-AT1G55580.1-R	CGTTAACGAAACCAAACCTCTT
q-AT2G36890.1-F	GACAAGGCAAATGTGAAGAGAG	q-AT2G36890.1-R	TTAACCATCTCAGTCTGCAACT
q-AT2G26170.1-F	ATGGCTAAGTATGGTCCTGATG	q-AT2G26170.1-R	TTTCTTGTGAAGAGGAGAAGCT
q-AT2G44990.1-F	GAATGTCACCAATGGTATCAGC	q-AT2G44990.1-R	AAATCGCCGTTATCCTCTCTAG
q-AT4G32810.1-F	CACTTTACAAGTTCGAGTGGTG	q-AT4G32810.1-R	ACAACAATCTGCAATGATGACC
q-AT3G18550-F1	TGACCATTATTCCTCACAACCA	q-AT3G18550-R	TGGGATGGTTTGGTTATCATCA
actin2-F	CCTTCGTCTTGATCTTGCGG	actin2-R	AGCGATGGCTGGAACAGAAC
**Primers for in situ probe**			
CsWOX9-SP6	GATTTAGGTGACACTATAGAATGCTTCCCAAACCACTACAACAACC	CsWOX9-T7	TGTAATACGACTCACTATAGGGAGAGGCGTGGAAATAGTGGA
